# 7-Iso­propyl­idene-*N* ^2^,*N* ^3^,*N* ^5^,*N* ^6^-tetra­meth­oxy-*N* ^2^,*N* ^3^,*N* ^5^,*N* ^6^-tetra­methylbi­cyclo­[2.2.1]hepta-2,5-diene-2,3,5,6-tetra­carboxamide

**DOI:** 10.1107/S1600536814004255

**Published:** 2014-03-15

**Authors:** Benjamin Sahlmann, Christian Näther, Rainer Herges

**Affiliations:** a Institut für Organische Chemie, Universität Kiel, Otto-Hahn-Platz 4, 24118 Kiel, Germany; b Institut für Anorganische Chemie, Universität Kiel, Otto-Hahn-Platz 6/7, 24118 Kiel, Germany

## Abstract

Although the mol­ecular structure of the title compound, C_22_H_32_N_4_O_8_, displays a twofold symmetry of the mol­ecule including the meth­oxy and methyl substituents, no crystallographic twofold symmetry is observed in the X-ray structure analysis. The carbonyl O atoms alternately point to different sides of the plane defined by the carbonyl C atoms. Two meth­oxy groups are oriented inside the mol­ecules cavity. The H atoms of two methyl groups are disordered over two orientations and were refined using a split model.

## Related literature   

For background to this work, see: Winkler *et al.* (2003*a* ▶, 2012 ▶). For the structure of 7-iso­propyl­idenenorborna-2,5-diene-2,3,5,6-tetra­carb­oxy­lic acid tetra­kis­(di­ethyl­amide), see: Winkler *et al.* (2003*b* ▶).
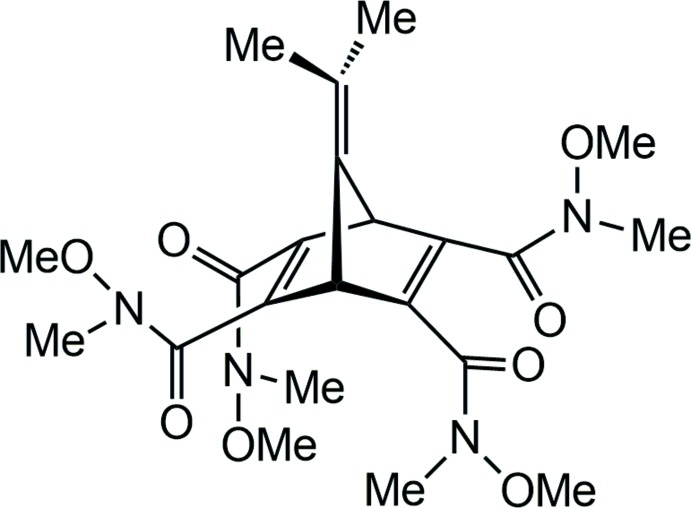


## Experimental   

### 

#### Crystal data  

C_22_H_32_N_4_O_8_
*M* *_r_* = 480.52
Triclinic, 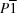

*a* = 9.5830 (9) Å
*b* = 10.2662 (8) Å
*c* = 13.3792 (16) Å
α = 94.648 (12)°
β = 91.548 (13)°
γ = 108.013 (10)°
*V* = 1245.7 (2) Å^3^
*Z* = 2
Mo *K*α radiation
μ = 0.10 mm^−1^
*T* = 200 K
0.3 × 0.3 × 0.2 mm

#### Data collection  

Stoe IPDS-1 diffractometer
10017 measured reflections
4788 independent reflections
3431 reflections with *I* > 2σ(*I*)
*R*_int_ = 0.040

#### Refinement  

*R*[*F* ^2^ > 2σ(*F* ^2^)] = 0.045
*wR*(*F* ^2^) = 0.119
*S* = 1.00
4788 reflections
318 parameters
H-atom parameters constrained
Δρ_max_ = 0.19 e Å^−3^
Δρ_min_ = −0.19 e Å^−3^

### 

Data collection: *X-AREA* (Stoe & Cie, 2008 ▶); cell refinement: *X-AREA*; data reduction: *X-AREA*; program(s) used to solve structure: *SHELXS97* (Sheldrick, 2008 ▶); program(s) used to refine structure: *SHELXL97* (Sheldrick, 2008 ▶); molecular graphics: *XP* in *SHELXTL* (Sheldrick, 2008 ▶); software used to prepare material for publication: XCIF in *SHELXTL*.

## Supplementary Material

Crystal structure: contains datablock(s) I, New_Global_Publ_Block. DOI: 10.1107/S1600536814004255/im2450sup1.cif

Structure factors: contains datablock(s) I. DOI: 10.1107/S1600536814004255/im2450Isup2.hkl

Supporting information file. DOI: 10.1107/S1600536814004255/im2450Isup3.cml

CCDC reference: 878002

Additional supporting information:  crystallographic information; 3D view; checkCIF report

## Figures and Tables

**Fig. 1.**
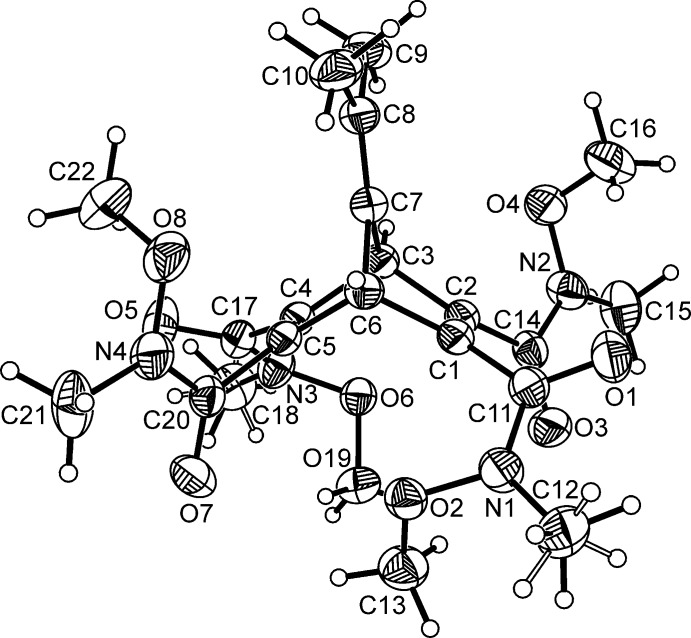
Crystal structure of the title compound with labeling and displacement ellipsoids drawn at the 50% probability level. Disordering is shown with full and open bonds.

## supplementary crystallographic information

### 1. Comment

The tertiary tetra amides of norbornadiene and quadricyclane are interesting compounds because of their ability to form stable complexes with alkali and alkaline earth metal cations. (Winkler *et al.*, 2003*a*; Winkler *et al.*, 2012) To enable further synthetic modifications, the tetrakis(*N*,*O*-dimethylhydroxyl amide) was synthesized. For the identification of this compound, a structure determination was performed.

In the structure of the title compound, the carbonyl atoms alternately point to different sides of the plane defined by the carbony C atoms. In contrast to the tetrakis(diethyl amide) (Winkler *et al.*, 2003*b*), the methoxy substituents are small enough to point inside of the cavity of the molecule. although the molecule displays twofold symmetry no crystallographic twofold symmetry is oberved experimentally. In addition, no intramolecular hydrogen bonds are found.

### 2. Experimental

7-Isopropylidenenorborna-2,5-diene-2,3,5,6-tetracarboxylic acid (250 mg, 0.81 mmol), suspended in anhydrous ethyl acetate (40 ml), was cooled to 0 °C and sonicated. After 5 min, diisopropyl ethylamine (2.20 ml, 13.0 mmol) was added and the mixture was sonicated for another 5 min. After the addition of *N*,*O*-dimethylhydroxylamine-hydrochloride (318 mg, 3.24 mmol) and *n*-propyl phosphonic acid anhydride (T3P) (2.43 ml, 4.05 mmol) (50% solution in ethyl acetate), the mixture was sonicated for 30 min and stirred at RT for 16 h. The mixture was then heated to reflux for 5 h. The reaction was stopped by addition of water (25 ml). After extraction with ethyl acetate, the combined organic phases were washed with 50 ml of brine and dried over magensium sulfate. After removal of the solvent, recrystallization from ethyl acetate afforded colorless crystals in 51% yield. mp.: 168 °C

### 3. Refinement

Hydrogen atoms were positioned with idealized geometry (methyl H atoms allowed to rotate but not to tip) and were refined isotropically with *U*_iso_(H) = 1.2 *U*_eq_(C)) (1.5 for methyl H atoms) using a riding model. The H atoms of two methyl groups are disordered and were refined using a split model with two orientations rotated by 60° and sof of 60:40.

### Crystal data

**Table:**

C_22_H_32_N_4_O_8_	*Z* = 2
*M**_r_* = 480.52	*F*(000) = 512
Triclinic, *P*1	*D*_x_ = 1.281 Mg m^−^^3^
Hall symbol: -P 1	Mo *K*α radiation, λ = 0.71073 Å
*a* = 9.5830 (9) Å	Cell parameters from 3915 reflections
*b* = 10.2662 (8) Å	θ = 4–28°
*c* = 13.3792 (16) Å	µ = 0.10 mm^−^^1^
α = 94.648 (12)°	*T* = 200 K
β = 91.548 (13)°	Block, colorless
γ = 108.013 (10)°	0.3 × 0.3 × 0.2 mm
*V* = 1245.7 (2) Å^3^	

### Data collection

**Table:**

Stoe IPDS-1 diffractometer	3431 reflections with *I* > 2σ(*I*)
Radiation source: fine-focus sealed tube	*R*_int_ = 0.040
Graphite monochromator	θ_max_ = 26.0°, θ_min_ = 2.9°
Phi scans	*h* = −11→11
10017 measured reflections	*k* = −12→12
4788 independent reflections	*l* = −16→16

### Refinement

**Table:**

Refinement on *F*^2^	Secondary atom site location: difference Fourier map
Least-squares matrix: full	Hydrogen site location: inferred from neighbouring sites
*R*[*F*^2^ > 2σ(*F*^2^)] = 0.045	H-atom parameters constrained
*wR*(*F*^2^) = 0.119	*w* = 1/[σ^2^(*F*_o_^2^) + (0.0701*P*)^2^] where *P* = (*F*_o_^2^ + 2*F*_c_^2^)/3
*S* = 1.00	(Δ/σ)_max_ < 0.001
4788 reflections	Δρ_max_ = 0.19 e Å^−^^3^
318 parameters	Δρ_min_ = −0.19 e Å^−^^3^
0 restraints	Extinction correction: *SHELXL97* (Sheldrick, 2008), Fc^*^=kFc[1+0.001xFc^2^λ^3^/sin(2θ)]^-1/4^
Primary atom site location: structure-invariant direct methods	Extinction coefficient: 0.079 (8)

### Special details

**Table:**

Experimental. ^1^H-NMR (500 MHz, 300 K, CDCl_3_, TMS): δ = 4.65 (s, 2H, H-1,4), 3.62 (s, 12H, H12), 3.25 (s, 12H, H-11), 1.53 (s, 6H, H-9) p.p.m.. ^13^C-NMR (125 MHz, 300 K, CDCl_3_, TMS): δ = 165.8 (C-10), 160.5 (C-7), 151.1 (C-2,3,5,6), 100.4 (C-8), 61.6 (C-12), 56.5 (C-1,4), 32.1 (C-11), 18.3 (C-9) p.p.m.. MS (EI, 70 eV): m/*z*(%)= 480 (2) [*M*]^+^, 421 (24), 420 (100), 329 (14), 328 (13), 269 (13), 268 (46), 240 (21), 220 (26). MS (CI, isobutane): m/*z*(%)= 481 (100) [*M*+H]^+^. UV/Vis (CHCl_3_): λ(_max_)(lg ε)= 262 nm (3.855), 314 nm (2.881).
Geometry. All e.s.d.'s (except the e.s.d. in the dihedral angle between two l.s. planes) are estimated using the full covariance matrix. The cell e.s.d.'s are taken into account individually in the estimation of e.s.d.'s in distances, angles and torsion angles; correlations between e.s.d.'s in cell parameters are only used when they are defined by crystal symmetry. An approximate (isotropic) treatment of cell e.s.d.'s is used for estimating e.s.d.'s involving l.s. planes.
Refinement. Refinement of *F*^2^ against ALL reflections. The weighted *R*-factor *wR* and goodness of fit *S* are based on *F*^2^, conventional *R*-factors *R* are based on *F*, with *F* set to zero for negative *F*^2^. The threshold expression of *F*^2^ > σ(*F*^2^) is used only for calculating *R*-factors(gt) *etc*. and is not relevant to the choice of reflections for refinement. *R*-factors based on *F*^2^ are statistically about twice as large as those based on *F*, and *R*- factors based on ALL data will be even larger.

### Fractional atomic coordinates and isotropic or equivalent isotropic displacement parameters (Å^2^)

**Table:**

	*x*	*y*	*z*	*U*_iso_*/*U*_eq_	Occ. (<1)
C1	0.81447 (18)	0.39743 (16)	0.81937 (12)	0.0298 (3)	
C2	0.67642 (18)	0.36772 (16)	0.78212 (13)	0.0300 (3)	
C3	0.63158 (18)	0.21966 (17)	0.73104 (13)	0.0308 (4)	
H3	0.5240	0.1707	0.7187	0.037*	
C4	0.72948 (19)	0.21953 (16)	0.64136 (13)	0.0301 (3)	
C5	0.86786 (18)	0.24874 (16)	0.67833 (12)	0.0295 (3)	
C6	0.86494 (18)	0.27007 (16)	0.79390 (13)	0.0302 (3)	
H6	0.9515	0.2636	0.8343	0.036*	
C7	0.71645 (19)	0.15921 (17)	0.80360 (13)	0.0319 (4)	
C8	0.6783 (2)	0.04726 (18)	0.85174 (13)	0.0386 (4)	
C9	0.5221 (3)	−0.0464 (2)	0.84862 (16)	0.0501 (5)	
H9A	0.4600	−0.0117	0.8055	0.075*	
H9B	0.4866	−0.0493	0.9167	0.075*	
H9C	0.5180	−0.1393	0.8217	0.075*	
C10	0.7892 (3)	0.0025 (2)	0.91079 (16)	0.0505 (5)	
H10A	0.8882	0.0643	0.9024	0.076*	
H10B	0.7832	−0.0917	0.8862	0.076*	
H10C	0.7682	0.0059	0.9821	0.076*	
C11	0.89827 (19)	0.52489 (18)	0.88315 (13)	0.0341 (4)	
O1	0.85560 (18)	0.55873 (16)	0.96400 (11)	0.0500 (4)	
N1	1.02489 (18)	0.60006 (17)	0.84779 (12)	0.0404 (4)	
C12	1.1278 (3)	0.7203 (2)	0.90131 (19)	0.0544 (5)	
H12A	1.0857	0.7446	0.9633	0.082*	0.60
H12B	1.1485	0.7971	0.8592	0.082*	0.60
H12C	1.2193	0.7010	0.9178	0.082*	0.60
H12D	1.2166	0.7505	0.8636	0.082*	0.40
H12E	1.1539	0.6980	0.9677	0.082*	0.40
H12F	1.0831	0.7942	0.9091	0.082*	0.40
O2	1.06466 (14)	0.55339 (13)	0.75543 (10)	0.0359 (3)	
C13	1.0318 (3)	0.6292 (2)	0.67700 (16)	0.0481 (5)	
H13A	0.9303	0.6306	0.6808	0.072*	
H13B	1.0437	0.5849	0.6115	0.072*	
H13C	1.0990	0.7237	0.6853	0.072*	
C14	0.58711 (19)	0.46339 (18)	0.78346 (13)	0.0335 (4)	
O3	0.63333 (18)	0.57977 (14)	0.75583 (12)	0.0502 (4)	
N2	0.45160 (17)	0.41573 (17)	0.81724 (13)	0.0403 (4)	
C15	0.3392 (2)	0.4826 (3)	0.81249 (18)	0.0535 (5)	
H15A	0.3831	0.5771	0.7954	0.080*	
H15B	0.2957	0.4839	0.8778	0.080*	
H15C	0.2627	0.4319	0.7610	0.080*	
O4	0.40977 (16)	0.28673 (14)	0.85165 (12)	0.0482 (4)	
C16	0.4368 (4)	0.3012 (3)	0.9592 (2)	0.0717 (8)	
H16A	0.5405	0.3524	0.9763	0.108*	
H16B	0.4134	0.2099	0.9837	0.108*	
H16C	0.3749	0.3512	0.9908	0.108*	
C17	0.6746 (2)	0.17624 (18)	0.53508 (13)	0.0354 (4)	
O5	0.7086 (2)	0.08738 (17)	0.48378 (11)	0.0580 (4)	
N3	0.57718 (19)	0.23375 (16)	0.49947 (12)	0.0396 (4)	
C18	0.5110 (3)	0.2035 (3)	0.39838 (16)	0.0542 (5)	
H18A	0.4217	0.2308	0.3958	0.081*	0.60
H18B	0.4859	0.1046	0.3786	0.081*	0.60
H18C	0.5807	0.2546	0.3522	0.081*	0.60
H18D	0.5705	0.1625	0.3553	0.081*	0.40
H18E	0.5063	0.2887	0.3725	0.081*	0.40
H18F	0.4115	0.1387	0.3989	0.081*	0.40
O6	0.56596 (14)	0.35176 (13)	0.55432 (10)	0.0365 (3)	
C19	0.6728 (2)	0.4717 (2)	0.52256 (16)	0.0458 (5)	
H19A	0.7707	0.4607	0.5275	0.069*	
H19B	0.6721	0.5533	0.5658	0.069*	
H19C	0.6484	0.4829	0.4528	0.069*	
C20	1.00122 (19)	0.26034 (17)	0.62073 (14)	0.0341 (4)	
O7	1.03798 (18)	0.33922 (16)	0.55533 (12)	0.0524 (4)	
N4	1.08090 (18)	0.18055 (17)	0.64597 (14)	0.0440 (4)	
C21	1.2220 (2)	0.1841 (3)	0.6077 (2)	0.0571 (6)	
H21A	1.2963	0.2065	0.6636	0.086*	
H21B	1.2503	0.2543	0.5601	0.086*	
H21C	1.2149	0.0940	0.5735	0.086*	
O8	1.02474 (18)	0.08363 (15)	0.71379 (11)	0.0482 (4)	
C22	0.9419 (3)	−0.0452 (2)	0.6592 (2)	0.0641 (7)	
H22A	0.8626	−0.0316	0.6176	0.096*	
H22B	0.8998	−0.1127	0.7067	0.096*	
H22C	1.0070	−0.0787	0.6160	0.096*	

### Atomic displacement parameters (Å^2^)

**Table:**

	*U*^11^	*U*^22^	*U*^33^	*U*^12^	*U*^13^	*U*^23^
C1	0.0281 (8)	0.0296 (8)	0.0321 (8)	0.0086 (6)	0.0069 (7)	0.0048 (6)
C2	0.0280 (8)	0.0302 (8)	0.0329 (8)	0.0100 (6)	0.0074 (7)	0.0046 (6)
C3	0.0276 (8)	0.0304 (8)	0.0345 (8)	0.0083 (6)	0.0046 (7)	0.0044 (6)
C4	0.0324 (8)	0.0270 (7)	0.0340 (8)	0.0126 (6)	0.0055 (7)	0.0057 (6)
C5	0.0325 (8)	0.0260 (7)	0.0343 (8)	0.0139 (6)	0.0072 (7)	0.0068 (6)
C6	0.0278 (8)	0.0298 (8)	0.0345 (8)	0.0103 (6)	0.0035 (7)	0.0063 (6)
C7	0.0342 (9)	0.0296 (8)	0.0327 (8)	0.0103 (7)	0.0049 (7)	0.0045 (6)
C8	0.0508 (11)	0.0304 (8)	0.0314 (8)	0.0079 (8)	0.0054 (8)	0.0028 (7)
C9	0.0607 (13)	0.0370 (9)	0.0391 (10)	−0.0051 (9)	0.0060 (9)	0.0051 (8)
C10	0.0727 (15)	0.0383 (10)	0.0409 (10)	0.0163 (10)	−0.0020 (10)	0.0119 (8)
C11	0.0319 (9)	0.0348 (8)	0.0361 (9)	0.0112 (7)	0.0035 (7)	0.0034 (7)
O1	0.0539 (9)	0.0508 (8)	0.0419 (8)	0.0134 (7)	0.0120 (7)	−0.0069 (6)
N1	0.0347 (8)	0.0413 (8)	0.0370 (8)	0.0016 (6)	0.0022 (7)	−0.0029 (6)
C12	0.0439 (12)	0.0444 (11)	0.0608 (13)	−0.0028 (9)	−0.0015 (10)	−0.0091 (10)
O2	0.0321 (6)	0.0378 (6)	0.0402 (7)	0.0133 (5)	0.0062 (5)	0.0065 (5)
C13	0.0540 (12)	0.0472 (11)	0.0474 (11)	0.0191 (9)	0.0053 (9)	0.0147 (9)
C14	0.0330 (9)	0.0386 (9)	0.0322 (8)	0.0157 (7)	0.0058 (7)	0.0043 (7)
O3	0.0551 (9)	0.0389 (7)	0.0660 (9)	0.0240 (6)	0.0220 (7)	0.0156 (7)
N2	0.0333 (8)	0.0482 (9)	0.0475 (9)	0.0210 (7)	0.0119 (7)	0.0148 (7)
C15	0.0409 (11)	0.0715 (14)	0.0590 (13)	0.0354 (11)	0.0030 (10)	−0.0013 (11)
O4	0.0421 (8)	0.0422 (7)	0.0591 (9)	0.0097 (6)	0.0181 (7)	0.0065 (6)
C16	0.100 (2)	0.0689 (16)	0.0577 (15)	0.0341 (15)	0.0352 (15)	0.0287 (13)
C17	0.0401 (9)	0.0314 (8)	0.0363 (9)	0.0137 (7)	0.0036 (7)	0.0015 (7)
O5	0.0851 (12)	0.0572 (9)	0.0439 (8)	0.0443 (9)	−0.0027 (8)	−0.0100 (7)
N3	0.0459 (9)	0.0369 (8)	0.0373 (8)	0.0169 (7)	−0.0064 (7)	−0.0024 (6)
C18	0.0593 (14)	0.0574 (13)	0.0419 (11)	0.0152 (11)	−0.0148 (10)	−0.0004 (9)
O6	0.0350 (7)	0.0360 (6)	0.0417 (7)	0.0153 (5)	0.0043 (5)	0.0050 (5)
C19	0.0511 (12)	0.0370 (9)	0.0491 (11)	0.0115 (8)	0.0054 (9)	0.0109 (8)
C20	0.0327 (9)	0.0320 (8)	0.0402 (9)	0.0140 (7)	0.0066 (7)	0.0007 (7)
O7	0.0570 (9)	0.0550 (9)	0.0540 (9)	0.0244 (7)	0.0276 (7)	0.0211 (7)
N4	0.0372 (9)	0.0429 (8)	0.0589 (10)	0.0215 (7)	0.0097 (8)	0.0075 (7)
C21	0.0368 (11)	0.0684 (14)	0.0708 (15)	0.0283 (10)	0.0042 (10)	−0.0142 (11)
O8	0.0591 (9)	0.0453 (8)	0.0506 (8)	0.0309 (7)	0.0012 (7)	0.0072 (6)
C22	0.0818 (18)	0.0351 (10)	0.0781 (17)	0.0223 (11)	0.0060 (14)	0.0040 (10)

### Geometric parameters (Å, º)

**Table:**

C1—C2	1.335 (3)	C14—O3	1.230 (2)
C1—C11	1.489 (2)	C14—N2	1.343 (2)
C1—C6	1.545 (2)	N2—O4	1.381 (2)
C2—C14	1.488 (2)	N2—C15	1.447 (2)
C2—C3	1.541 (2)	C15—H15A	0.9800
C3—C7	1.535 (2)	C15—H15B	0.9800
C3—C4	1.543 (2)	C15—H15C	0.9800
C3—H3	1.0000	O4—C16	1.443 (3)
C4—C5	1.337 (3)	C16—H16A	0.9800
C4—C17	1.486 (3)	C16—H16B	0.9800
C5—C20	1.488 (2)	C16—H16C	0.9800
C5—C6	1.546 (2)	C17—O5	1.226 (2)
C6—C7	1.539 (2)	C17—N3	1.345 (2)
C6—H6	1.0000	N3—O6	1.400 (2)
C7—C8	1.320 (2)	N3—C18	1.446 (3)
C8—C9	1.506 (3)	C18—H18A	0.9800
C8—C10	1.510 (3)	C18—H18B	0.9800
C9—H9A	0.9800	C18—H18C	0.9800
C9—H9B	0.9800	C18—H18D	0.9800
C9—H9C	0.9800	C18—H18E	0.9800
C10—H10A	0.9800	C18—H18F	0.9800
C10—H10B	0.9800	O6—C19	1.440 (2)
C10—H10C	0.9800	C19—H19A	0.9800
C11—O1	1.228 (2)	C19—H19B	0.9800
C11—N1	1.343 (2)	C19—H19C	0.9800
N1—O2	1.394 (2)	C20—O7	1.225 (2)
N1—C12	1.441 (3)	C20—N4	1.336 (2)
C12—H12A	0.9800	N4—O8	1.394 (2)
C12—H12B	0.9800	N4—C21	1.450 (2)
C12—H12C	0.9800	C21—H21A	0.9800
C12—H12D	0.9800	C21—H21B	0.9800
C12—H12E	0.9800	C21—H21C	0.9800
C12—H12F	0.9800	O8—C22	1.441 (3)
O2—C13	1.440 (2)	C22—H22A	0.9800
C13—H13A	0.9800	C22—H22B	0.9800
C13—H13B	0.9800	C22—H22C	0.9800
C13—H13C	0.9800		
			
C2—C1—C11	125.72 (15)	H13A—C13—H13C	109.5
C2—C1—C6	107.71 (14)	H13B—C13—H13C	109.5
C11—C1—C6	126.28 (15)	O3—C14—N2	121.23 (16)
C1—C2—C14	126.22 (16)	O3—C14—C2	122.67 (15)
C1—C2—C3	107.02 (14)	N2—C14—C2	116.10 (15)
C14—C2—C3	126.62 (15)	C14—N2—O4	118.04 (14)
C7—C3—C2	98.31 (13)	C14—N2—C15	125.53 (17)
C7—C3—C4	96.76 (12)	O4—N2—C15	116.18 (16)
C2—C3—C4	107.50 (13)	N2—C15—H15A	109.5
C7—C3—H3	117.0	N2—C15—H15B	109.5
C2—C3—H3	117.0	H15A—C15—H15B	109.5
C4—C3—H3	117.0	N2—C15—H15C	109.5
C5—C4—C17	126.88 (15)	H15A—C15—H15C	109.5
C5—C4—C3	107.59 (15)	H15B—C15—H15C	109.5
C17—C4—C3	125.07 (15)	N2—O4—C16	108.98 (17)
C4—C5—C20	127.32 (16)	O4—C16—H16A	109.5
C4—C5—C6	107.01 (14)	O4—C16—H16B	109.5
C20—C5—C6	125.67 (15)	H16A—C16—H16B	109.5
C7—C6—C1	97.69 (12)	O4—C16—H16C	109.5
C7—C6—C5	96.70 (13)	H16A—C16—H16C	109.5
C1—C6—C5	107.58 (12)	H16B—C16—H16C	109.5
C7—C6—H6	117.2	O5—C17—N3	121.17 (18)
C1—C6—H6	117.2	O5—C17—C4	122.52 (16)
C5—C6—H6	117.2	N3—C17—C4	116.18 (15)
C8—C7—C3	132.86 (17)	C17—N3—O6	117.33 (15)
C8—C7—C6	132.60 (17)	C17—N3—C18	124.05 (17)
C3—C7—C6	94.40 (13)	O6—N3—C18	116.83 (15)
C7—C8—C9	122.14 (19)	N3—C18—H18A	109.5
C7—C8—C10	122.15 (19)	N3—C18—H18B	109.5
C9—C8—C10	115.68 (17)	H18A—C18—H18B	109.5
C8—C9—H9A	109.5	N3—C18—H18C	109.5
C8—C9—H9B	109.5	H18A—C18—H18C	109.5
H9A—C9—H9B	109.5	H18B—C18—H18C	109.5
C8—C9—H9C	109.5	N3—C18—H18D	109.5
H9A—C9—H9C	109.5	N3—C18—H18E	109.5
H9B—C9—H9C	109.5	H18D—C18—H18E	109.5
C8—C10—H10A	109.5	N3—C18—H18F	109.5
C8—C10—H10B	109.5	H18D—C18—H18F	109.5
H10A—C10—H10B	109.5	H18E—C18—H18F	109.5
C8—C10—H10C	109.5	N3—O6—C19	109.55 (14)
H10A—C10—H10C	109.5	O6—C19—H19A	109.5
H10B—C10—H10C	109.5	O6—C19—H19B	109.5
O1—C11—N1	121.74 (18)	H19A—C19—H19B	109.5
O1—C11—C1	122.24 (16)	O6—C19—H19C	109.5
N1—C11—C1	116.02 (15)	H19A—C19—H19C	109.5
C11—N1—O2	117.70 (15)	H19B—C19—H19C	109.5
C11—N1—C12	124.80 (17)	O7—C20—N4	121.58 (16)
O2—N1—C12	117.35 (15)	O7—C20—C5	122.68 (15)
N1—C12—H12A	109.5	N4—C20—C5	115.73 (15)
N1—C12—H12B	109.5	C20—N4—O8	118.28 (15)
H12A—C12—H12B	109.5	C20—N4—C21	125.56 (18)
N1—C12—H12C	109.5	O8—N4—C21	116.16 (17)
H12A—C12—H12C	109.5	N4—C21—H21A	109.5
H12B—C12—H12C	109.5	N4—C21—H21B	109.5
N1—C12—H12D	109.5	H21A—C21—H21B	109.5
N1—C12—H12E	109.5	N4—C21—H21C	109.5
H12D—C12—H12E	109.5	H21A—C21—H21C	109.5
N1—C12—H12F	109.5	H21B—C21—H21C	109.5
H12D—C12—H12F	109.5	N4—O8—C22	109.25 (17)
H12E—C12—H12F	109.5	O8—C22—H22A	109.5
N1—O2—C13	110.50 (14)	O8—C22—H22B	109.5
O2—C13—H13A	109.5	H22A—C22—H22B	109.5
O2—C13—H13B	109.5	O8—C22—H22C	109.5
H13A—C13—H13B	109.5	H22A—C22—H22C	109.5
O2—C13—H13C	109.5	H22B—C22—H22C	109.5
